# Effects and Physiological Mechanism of Foliar Zinc‐Fulvic Acid Spraying on Cadmium and Zinc Accumulation in Rice (*Oryza sativa L.*): Insights From Metabolomics Analysis

**DOI:** 10.1002/fsn3.70391

**Published:** 2025-06-13

**Authors:** Yuhua Yang, Xianping Yang, Zhidong Xu, Qinhui Lu, Qinghai Zhang

**Affiliations:** ^1^ School of Public Health, the Key Laboratory of Environmental Pollution Monitoring and Disease Control Ministry of Education, Guizhou Medical University Guiyang China; ^2^ State Key Laboratory of Environmental Geochemistry, Institute of Geochemistry, Chinese Academy of Sciences Guiyang China

**Keywords:** cadmium, foliar application, metabolomics, rice, zinc

## Abstract

Although zinc‐fulvic acid chelate (Zn‐FA) can efficiently reduce the cadmium (Cd) accumulation in rice grains, little is known about the impacts on other beneficial elements (such as zinc) levels in rice, along with the optimal concentration and underlying molecular mechanisms. The aim of this study is to investigate the effect of Zn‐FA on Cd accumulation in rice and its metabolic mechanism through pot experiments. The results demonstrated that Zn‐FA treatments significantly reduced Cd and increased Zn content in rice grains, with 10 g/L Zn‐FA identified as the optimal concentration for spraying. Zn‐FA treatment primarily reduced the translocation of Cd from stems to grains, thereby limiting Cd accumulation in the grain. Metabolomic analysis revealed that Zn‐FA application significantly altered the metabolic profiles of rice leaves under Cd stress. In Yixiangyou586 (YXY586), Zn‐FA involved the upregulation of flavonoids with antioxidant properties, along with associated pathways such as flavonoid biosynthesis, flavone, and flavonol biosynthesis; similarly, in Gengxiangyou703 (GXY703), Zn‐FA modulated the metabolism of flavonoids. Under Zn‐FA application, the reduction of Cd content and increase of Zn accumulation in rice grains are mainly related to the altered metabolism of phenolics, especially flavonoids in rice leaves. The findings of this study offer new insights into the mechanism of Zn‐FA in reducing Cd content in rice, thereby providing a scientific foundation and technical support for ensuring safe crop production and promoting ecological safety.

## Graphical abstract

Foliar Zn‐FA application reduced Cd and increased Zn concentration in the grain. Foliar Zn‐FA application inhibited the translocation of Cd from stems to grains. Foliar Zn‐FA application significantly alters flavonoid and phenolic metabolites.

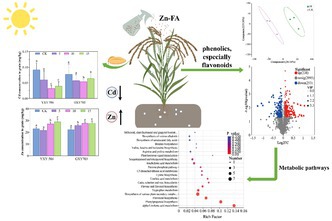

## 1 Introduction

Cadmium (Cd) is a toxic heavy metal that poses substantial risks to both plants and human health (Guo et al. 2021). Anthropogenic activities, such as mining, smelting, and fertilizer application, have contributed to Cd contamination in agricultural soils, presenting a global environmental concern (Zhao et al. 2014). In contrast, zinc (Zn) is an essential micronutrient and plays a crucial role in maintaining human health. In China and Southeast Asia, rice, a staple food crop, is a major source of both Zn and Cd in human diets (Jena et al. 2023; Chasapis et al. 2020; Yang et al. 2020). Excessive Cd intake can lead to severe health consequences, such as skeletal damage, kidney disease, and increased cancer risk (Xue et al. 2023). Similarly, Zn deficiency in the diet can also lead to various health issues (Jena et al. 2023; Wang et al. 2021), such as stunted growth, loss of appetite, impaired immune function, DNA damage, and an elevated risk of cancer (Liu et al. 2024; Wessels and Rink 2020). Consequently, reducing Cd concentrations while increasing Zn levels in rice grains is essential for improving human health and nutrition.

Recently, various physical, chemical, and biological remediation methods have been developed to mitigate Cd contamination in rice. Although these methods are effective, they often involve significant drawbacks, such as high remediation costs and the potential for secondary environmental pollution (Song et al. 2023). In contrast, foliar application technology has garnered considerable attention due to its effectiveness, safety, and cost‐efficiency (Wang, Liu, et al. 2024). Numerous studies have confirmed that foliar spraying with EDTA‐Zn or ZnSO_4_ significantly reduces Cd content in rice (Zheng et al. 2023; Wang et al. 2020). However, excessive foliar application of ZnSO_4_ can result in adverse effects, such as leaf burn, incomplete panicle development, and reduced grain yield (Xu et al. 2022). Furthermore, the exogenous application of chemical chelating agents like EDTA may negatively impact plant growth and soil quality (Wang et al. 2020; Han et al. 2020).

Humic substances, recognized as a cost‐effective and environmentally friendly soil amendment, have been shown to effectively reduce Cd absorption and accumulation in rice grains (Yu et al. 2018). Fulvic acid (FA), a component of humic acid, has a lower molecular weight and a higher number of oxygen‐containing functional groups (e.g., hydroxyl, carboxyl, phenolic, quinone, carbonyl, and semiquinone groups) compared to humic acid. These functional groups can bind to heavy metals, forming complexes that decrease heavy metal uptake by plants (Gao et al. 2022; Wang et al. 2019). For example, foliar spraying FA promotes growth under Cd stress while simultaneously reducing Cd content in lettuce tissues (Wang et al. 2019). Additionally, our previous study demonstrated that Zn‐FA is a highly effective organic chelated Zn compound in reducing Cd concentration in rice grains (Lu et al. 2024). However, its impact on Zn levels in rice, along with the optimal concentration and underlying molecular mechanisms, remains unclear.

In recent years, the application of plant metabolomics to study the metabolic response of plants under heavy metal stress has garnered increasing attention. Wu et al. (2024) and others found that Cd exposure induces metabolomic changes in rice leaves, where the contents of amino acids, polysaccharides, and proteins decrease with the increasing of exposure concentrations, disrupting 28 metabolic pathways, and consequently affecting the yield and quality of the crop. Therefore, this study will utilize metabolomics approaches to investigate the effects of Zn‐FA on foliar metabolite changes in rice, thereby elucidating the mechanisms of Cd accumulation in rice.

In light of these knowledge gaps, it is hypothesized that Zn‐FA treatment inhibits the translocation of Cd to the grain by modulating the synthesis of foliar metabolites, while promoting the partitioning of Zn. The primary objectives of this study are (1) to investigate the optimal concentration of Zn‐FA for foliar application, (2) to evaluate the impact of Zn‐FA on the accumulation of Cd and Zn in rice, and (3) to investigate its influence on foliar metabolites. This study aims to develop a promising method that simultaneously reduces Cd levels and enhances Zn content in rice, while elucidating the potential mechanisms via a metabolomics approach.

## 2 Materials and Methods

### 2.1 Experiment Design

The contaminated soil used for the pot experiment was collected from a Cd‐contaminated rice paddy field in the Tianzhu Ba mining region in Guizhou Province, China (26°41′50″N, 108°54′10″E). Soil pH was 6.7 ± 0.09. The soil was naturally air‐dried, sieved, and thoroughly mixed prior to use. Five kilograms of Cd‐contaminated paddy soil were placed in plastic pots and subjected to a 10‐day waterlogging treatment. According to our previous research (Lu et al. 2024), foliar spraying of 10 g/L Zn‐FA can significantly reduce the accumulation of Cd in rice. Thus, the pots were divided into four treatment groups: a control group (CK, sprayed with deionized water) and three Zn‐FA treatment groups (sprayed with Zn‐FA solutions at concentrations of 5, 10, and 15 g/L), with three replicates per group. Two widely cultivated local rice varieties, Yixiangyou586 (YXY586) and Gengxiangyou703 (GXY703), were selected for seedling cultivation. Uniformly growing rice seedlings were chosen for transplantation, with two seedlings planted per pot. Foliar spraying was conducted during the tillering, heading, and grain‐filling stages of rice growth using a pneumatic spray can. The dosage of each treatment was 200 mL at the tillering stage and 300 mL at the spiking and filling stages. We used plastic film to cover the soil surface during chemical spraying to reduce the contact between the chemical and the soil. To enhance the adhesion of the solution onto the leaves, five to eight drops of Tween 80 were added. Spraying was performed on windless, rainless days after 4:00 PM to ensure that the rice leaves were evenly moistened.

The water depth in the pots was maintained at approximately 2 cm throughout the rice growth period. Approximately 0.5 g urea and 0.5 g compound fertilizer were applied to each pot at the seedling stage, and 1.0 g urea and 1.2 g compound fertilizer were also applied to each pot at the tillering, spiking, and grouting stages, respectively. The pot experiment was conducted at Guizhou Medical University (26°22′31.29″ N, 106°38′18.38″ E).

### 2.2 Sample Collection and Determination

After the rice matured, three fresh leaves with fully expanded tips were collected, cleaned with deionized water, placed in a cooler of dry ice, and immediately transferred to a refrigerator at −80°C for preservation for metabolomics analysis. The grains, roots, stems, and leaves of the whole rice plant were collected separately, cleaned with deionized water, and brought back to the laboratory for processing. The samples were first subjected to 30 min of quenching at 105°C, followed by drying at 60°C until reaching a constant weight. The husks and brown rice were separated using a small hulling machine. The roots, stems, leaves, and rice grain samples were subsequently ground into powder, then filtered through a 100‐mesh sieve, and stored for analysis. Soil samples were air‐dried, ground using a ceramic mortar, processed through a 100‐mesh sieve, and stored for subsequent use.

The sample digestion procedures followed the protocol established by Lu et al. (2024). For plant samples, 0.1 g of the sample was digested with 3 mL of HNO_3_. Subsequently, the sample was digested at 158°C for 16 h. One milliliter of 30% H_2_O_2_ was added to the mixture and then heated on a heating plate at 95°C for 1 h. The temperature was subsequently increased to 105°C to evaporate the acid. For the soil sample, 0.05 g of the sample was weighed into a Teflon crucible, digested with 3 mL of HNO_3_ and 1 mL of HF, and then digested at 158°C for 48 h. One milliliter of HNO_3_ was added to the mixture and then evaporated on a hot plate at 105°C. All solutions were transferred and diluted for analysis. The concentrations of Cd and Zn were measured by an inductively coupled plasma mass spectrometer (NexION 2000, PerkinElmer Co. Ltd., USA).

During analysis, method blanks, certified reference materials, and duplicate samples were utilized for quality assurance and quality control. The relative standard deviation (RSD) of duplicates is less than 5%. Certified materials of citrus leaves (GBW10020) and yellow‐red soil (GSS‐5a) were used for plant and soil samples, with recoveries ranging from 106.9% to 116.9% and 87.2% to 98.3%, respectively.

### 2.3 Calculation of Transfer Factors

In this study, the transfer factors (TFs) of Cd and Zn from roots to grains, stems to grains, leaves to grains, and stems to leaves were calculated according to the method outlined by Zheng et al. (2023). The specific calculation formulas are as follows:

$${\mathrm{TF}}_{\text{root}-\text{grain}}=C_{\text{grain}}/C_{\text{root}}$$

$${\mathrm{TF}}_{\text{stem}-\text{grain}}=C_{\text{grain}}/C_{\text{stem}}$$

$${\mathrm{TF}}_{\text{leaf}-\text{grain}}=C_{\text{grain}}/C_{\text{leaf}}$$

$${\mathrm{TF}}_{\text{stem}-\text{leaf}}=C_{\text{leaf}}/C_{\text{stem}}$$

In these formulas, *C*_*grain*_, *C*_*root*_, *C*_*stem*_, and *C*_*leaf*_ represent the concentrations of Cd or Zn in grains, roots, stems, and leaves, respectively.

### 2.4 Statistical Analysis

In this study, data was processed with Microsoft Excel 2019. All data were expressed as mean ± standard deviation ($\bar{x}$ ± s, *n* = 3), one‐way analysis of variance (ANOVA) and graphing were performed using GraphPad Prism 9.5.0 software with a significance level of 0.05.

### 2.5 Untargeted Metabolomics Analysis

#### 2.5.1 Sample Extraction

A 100 mg rice leaf sample was added to a 2 mL centrifuge tube and a 6 mm diameter grinding bead was added. Subsequently, 800 μL of extractant solution (methanol: water = 4:1 v:v) was added, followed by ultrasonic extraction at low temperature for 30 min. The samples were then placed at −20°C for 30 min, after which they were centrifuged for 15 min (4°C, 13,000 g). The supernatant was transferred to the injection vial for LC–MS/MS analysis, which was conducted on a Thermo UHPLC‐Q Exactive system at Majorbio Bio‐Pharm Technology Co. Ltd. (Shanghai, China). The chromatographic separation was conducted using an ACQUITY UPLC BEH C18 column (100 mm × 2.1 mm i.d., 1.7 μm; Waters, USA) with a mobile phase consisting of solvent A containing 2% acetonitrile in water (containing 0.1% formic acid) and solvent B consisting of acetonitrile (containing 0.1% formic acid). The injection volume was 3 μL, the column temperature was set at 40°C, and the flow rate was set at 0.4 mL/min. The gradient elution procedures were as outlined below: 0–0.5 min, 2% solvent B; 0.5–7.5 min, 2%–35% solvent B; 7.5–13 min, 35%–95% solvent B; 13–14.4 min, 95% solvent B; 14.4–14.5 min, 95%–2% solvent B; 14.5–16 min, 2% solvent B. Both positive and negative ion modes were utilized during mass spectrometry analysis, with scan ranges from 70 to 1050 m/z and voltages of 3500 V and 3000 V, respectively. Throughout the instrumental analysis, the quality control sample (QC), prepared by mixing equal volumes of all samples, was inserted after every 12 analytical samples to monitor the stability of the analysis.

#### 2.5.2 Metabolomics Data Analysis

The LC/MS raw data were baseline filtered, integrated, peaks identified, aligned, and retention times corrected using Progenesis QI software (Waters, Milford, USA), and the MS and MS/MS mass spectral information was then matched against the self‐built‐specific metabolite database (MJDBPM, Shanghai Majorbio, China) to obtain metabolite information. All features with standard deviations greater than 30% based on QC samples were excluded to eliminate interference. The data were then evaluated using principal component analysis (PCA) and partial least squares discriminant analysis (PLS‐DA) by R package “ropls (version 1.6.2).” Reference to previous research methods, using Student's test, metabolites with Variable importance in the projection (VIP) > 1 and *p*‐values < 0.05 were considered as significantly different metabolites (Wang, Mu, et al. 2024; Wang, Mu, et al. 2023; Xiong et al. 2023). Differential metabolite annotation of metabolic pathways was performed through the Kyoto Encyclopedia of Genes and Genomes (KEGG) database. These metabolites were classified according to their biological functions pr involvement in metabolic pathways. Pathway enrichment analysis was conducted using the Python package “scipy stats” and the most relevant biological pathways to the experimental treatments were identified by Fisher's exact test.

## 3 Results

### 3.1 Effects of Spraying Zn‐FA on Cd Accumulation in Rice Tissue

**FIGURE 1:**
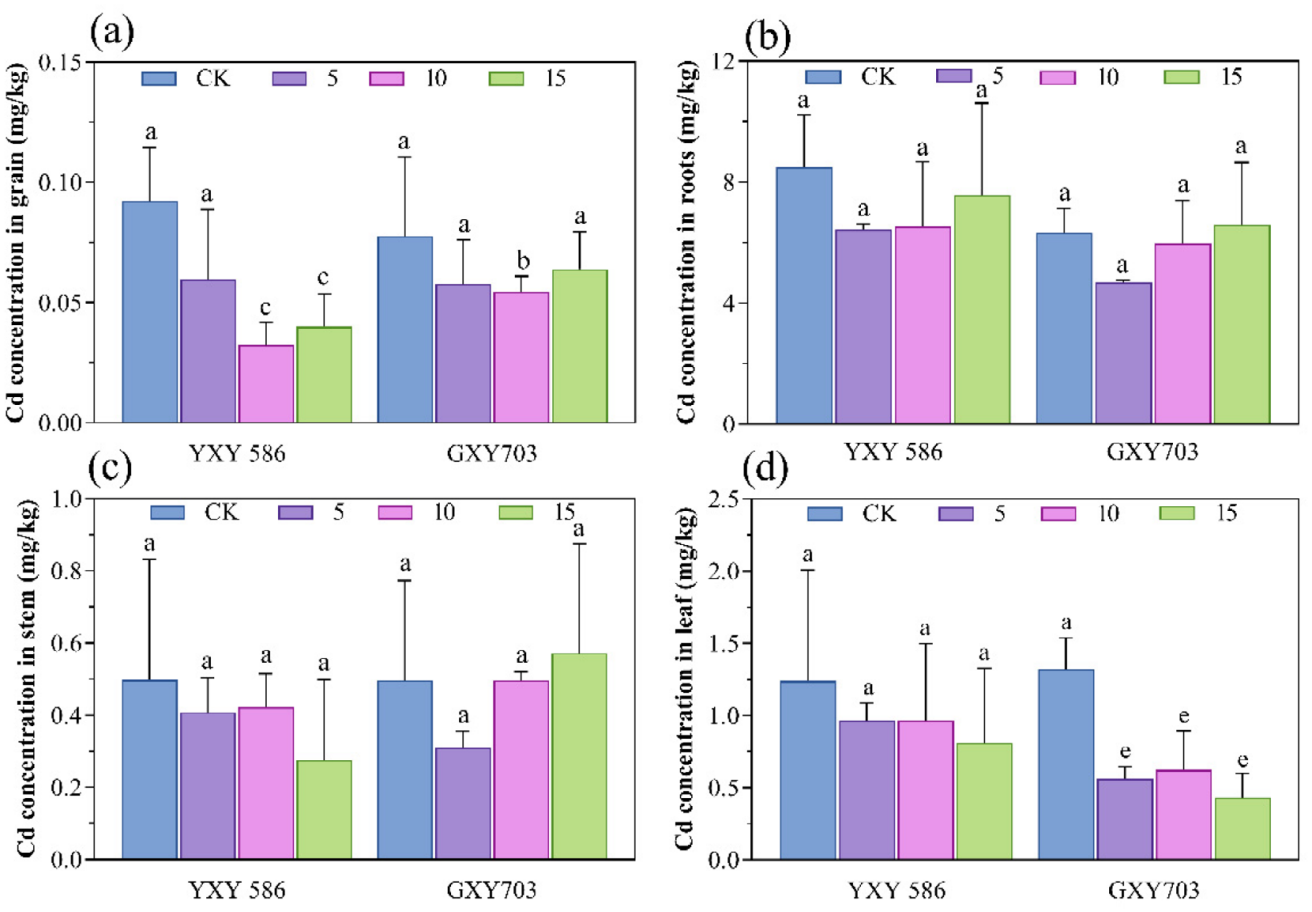
Effect of foliar application Zn‐FA on Cd concentrations in rice of YXY586 and GXY703 cultivars. The symbols (a), (b), (c), and (d) are Cd concentrations in grains, roots, stems, and leaves, respectively. CK stands for the control group, and 5, 10, and 15 for Zn‐FA treatment concentrations (g/L), respectively. The letters indicate significant differences between the control and the three treatment groups (*n* = 3, *p* < 0.05).

The effects of foliar application on Zn‐FA reduced the Cd content in rice grains for both rice varieties, as shown in Figure 1. For YXY586 (Figure 1a), Cd content in rice grains decreased by 34.1%, 64.8%, and 56.0% under treatments with 5, 10, and 15 g/L Zn‐FA, respectively, with all treated samples exhibiting Cd levels below China's limit for rice (0.2 mg/kg). The greatest reduction in Cd content was observed at the concentration of 10 g/L relative to the control group. Additionally, Zn‐FA application influenced Cd absorption in the roots, stems, and leaves of rice. Under the treatments of 5, 10, and 15 g/L Zn‐FA, the Cd content decreased by 24.3%, 23.1%, and 10.8% in the roots; 16.3%, 14.3%, and 44.9% in the stems; and 21.7%, 21.7%, and 34.8% in the leaves, respectively. For GXY703 (Figure 1a), the Cd content in rice grains decreased by 25.6%, 32.1%, and 17.9% under treatments with 5, 10, and 15 g/L Zn‐FA, respectively, relative to the control group. The most pronounced decrease in Cd content in grains was achieved at a concentration of 10 g/L. In this variety, Cd content in the roots and stems was lower than in the control group at 5 g/L Zn‐FA but increased at higher concentrations. Cd content in the leaves showed a significant reduction under all three treatment concentrations (*p* < 0.0001), decreasing by 59.4%, 53.6%, and 68.8%, respectively, compared to the control group.

### 3.2 Effects of Spraying Zn‐FA on Zn Accumulation in Rice Tissue

**FIGURE 2:**
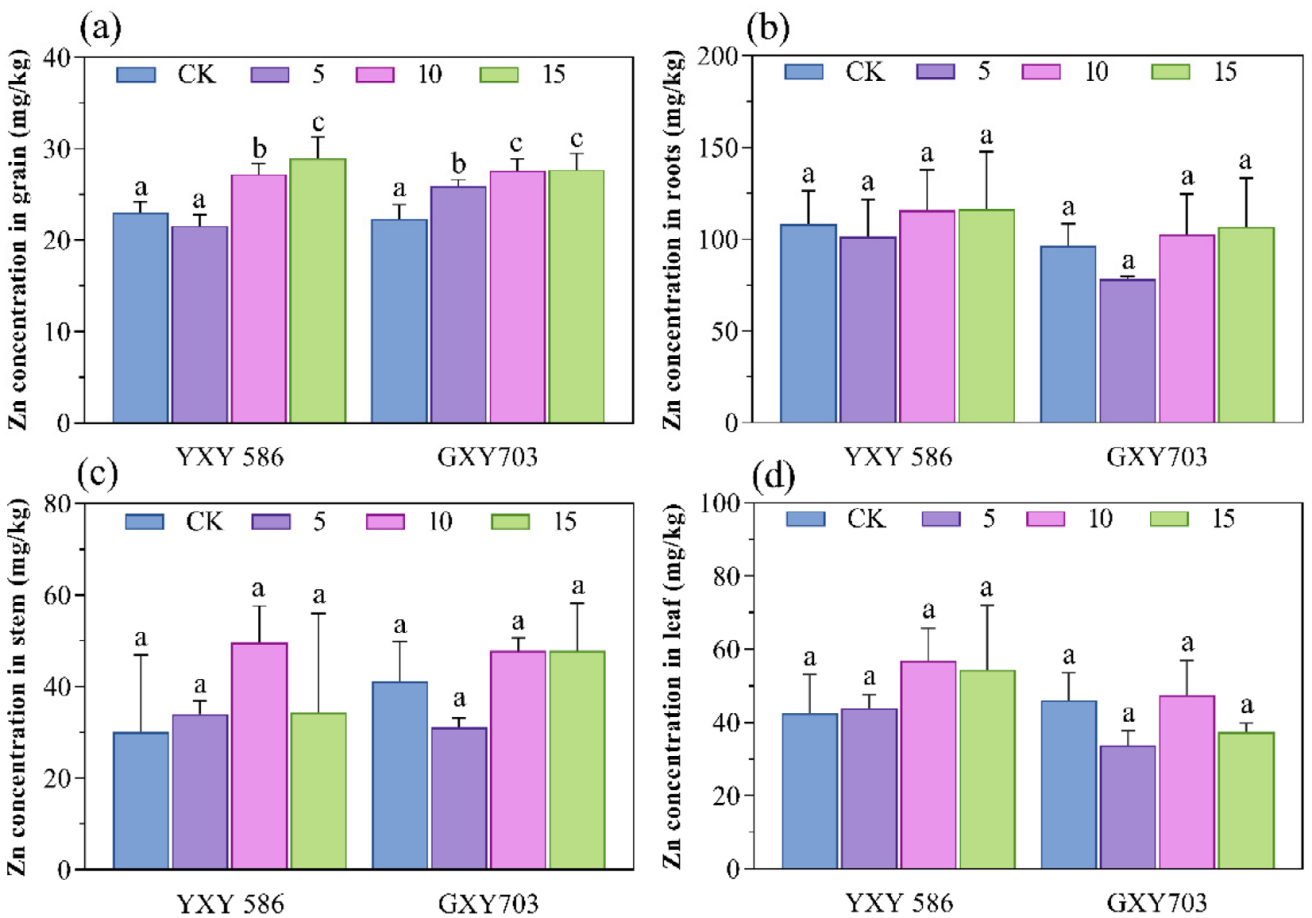
Effect of foliar application Zn‐FA on Zn concentrations in rice of YXY586 and GXY703 cultivars. The symbols (a), (b), (c), and (d) represent Zn concentrations in grains, roots, stems, and leaves, respectively. CK stands for the control group, and 5, 10, and 15 for Zn‐FA treatment concentrations (g/L), respectively. The letters indicate significant differences between the control and the three treatment groups (*n* = 3, *p* < 0.05).

The effect of foliar application of Zn‐FA on Zn content in rice tissues is shown in Figure 2. In the YXY586 variety, compared to the control group, Zn content in rice grains increased by 18.3% and 25.7% under 10 and 15 g/L Zn‐FA treatments, respectively, while the Zn content in rice grains decreased by 6.1% under 5 g/L Zn‐FA treatment. Moreover, under three concentration treatments, the Zn content in stems increased by 12.9%, 65.1%, and 14.2%, followed by increases of 3.2%, 33.6%, and 28.0% in leaves. In the GXY 703 variety, relative to the control group, Zn content in rice grains increased by 15.6%, 23.7%, and 24.4% under 5, 10, and 15 g/L Zn‐FA treatments, respectively. The Zn content in roots and stems of rice also increased under the 10 and 15 g/L treatments, with Zn content increasing by 7.4% and 11.6% in roots, followed by a higher increase of 16.3% in stems in both treatments.

### 3.3 The Impact of Foliar Application of Zn‐FA on Metal Translocation Factors

**FIGURE 3:**
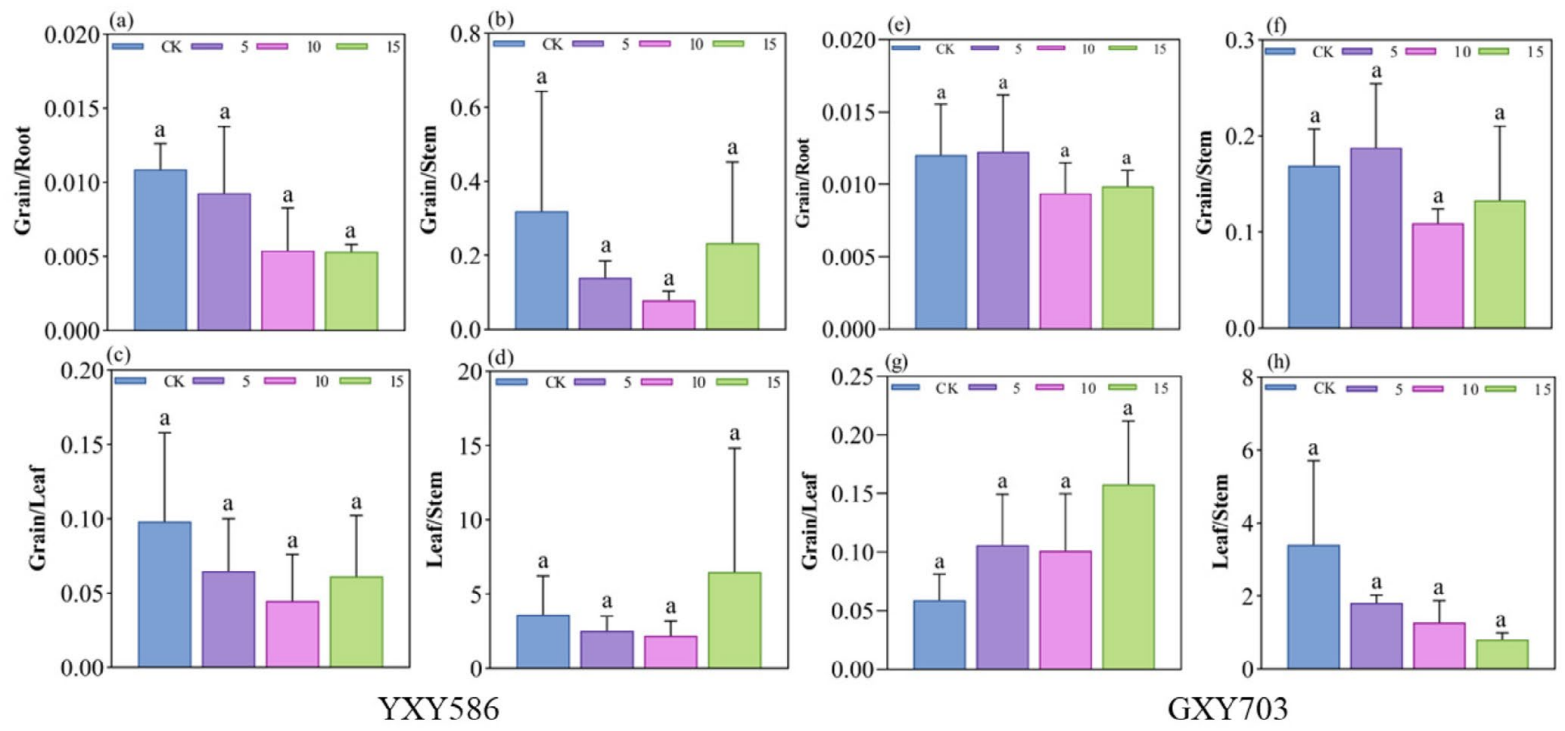
Effect of Zn‐FA application on Cd translocation factors. Root to grain (a), stem to grain (b), leaf to grain (c), stem to leaf (d) for YXY586; root to grain (e), stem to grain (f), leaf to grain (g), stem to leaf (h) for GXY703. CK stands for control group, and 5, 10, and 15 for Zn‐FA treatment concentrations (g/L), respectively. The letters indicate significant differences between the control and the three treatment groups (*n* = 3, *p* < 0.05).

The calculated transfer factor (TF) values are presented in Figure 3. It clearly indicates that Zn‐FA treatment influenced Cd translocation in both rice varieties, but the impact on Zn translocation within the organs of both rice varieties was not significant (Figure S1). In the YXY586 variety, compared to the control group, the primary reduction in Cd translocation was observed from roots, stems, and leaves to the rice grains, resulting in a decrease in Cd content in the rice grains. In the GXY703 variety, the primary reduction in Cd translocation occurred from stems to leaves. Moreover, at concentrations of 10 and 15 g/L, Zn‐FA significantly inhibited Cd translocation from roots and stems to rice grains.

### 3.4 Metabolomics Analysis

**FIGURE 4:**
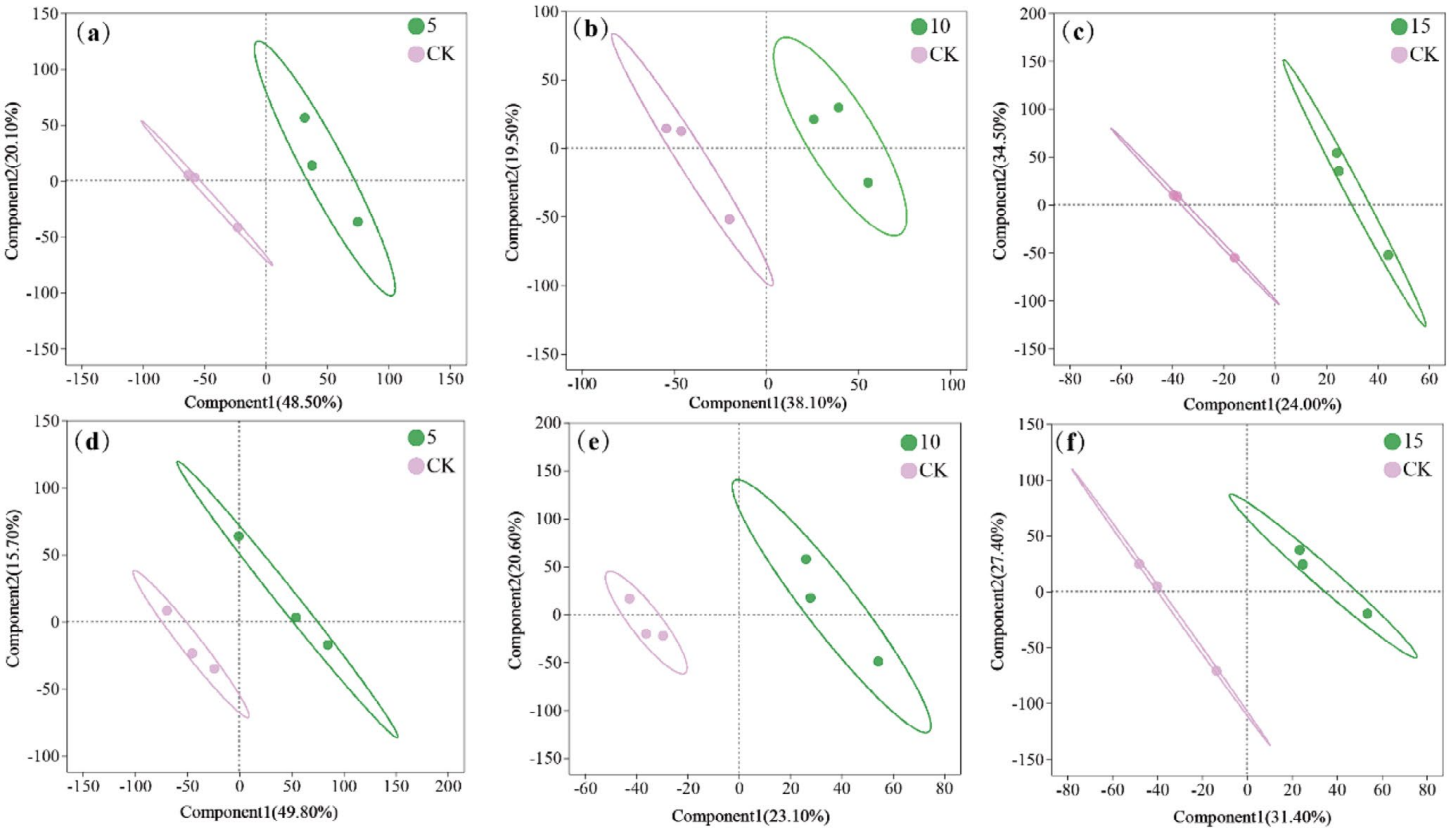
Partial least squares (PLS‐DA) scores plot. The symbols of (a), (b), and (c) represent YXY586 under the treatment concentrations of 5, 10, and 15 g/L Zn‐FA, respectively; and (d), (e), and (f) represent GXY703 under the treatment of 5, 10, and 15 g/L Zn‐FA, respectively.

Based on partial least squares discriminant analysis (PLS‐DA), a clear separation was observed between foliar application of different reagent concentrations and the control group for both varieties (YXY586 and GXY703) (Figure 4). This finding indicates that Zn‐FA application had an impact on the leaf metabolites of rice. Using the criteria of VIP scores (VIP > 1) from the OPLS‐DA model as well as the Student's t‐test (*p* < 0.05), a total of 984 differential metabolites were identified.

**FIGURE 5:**
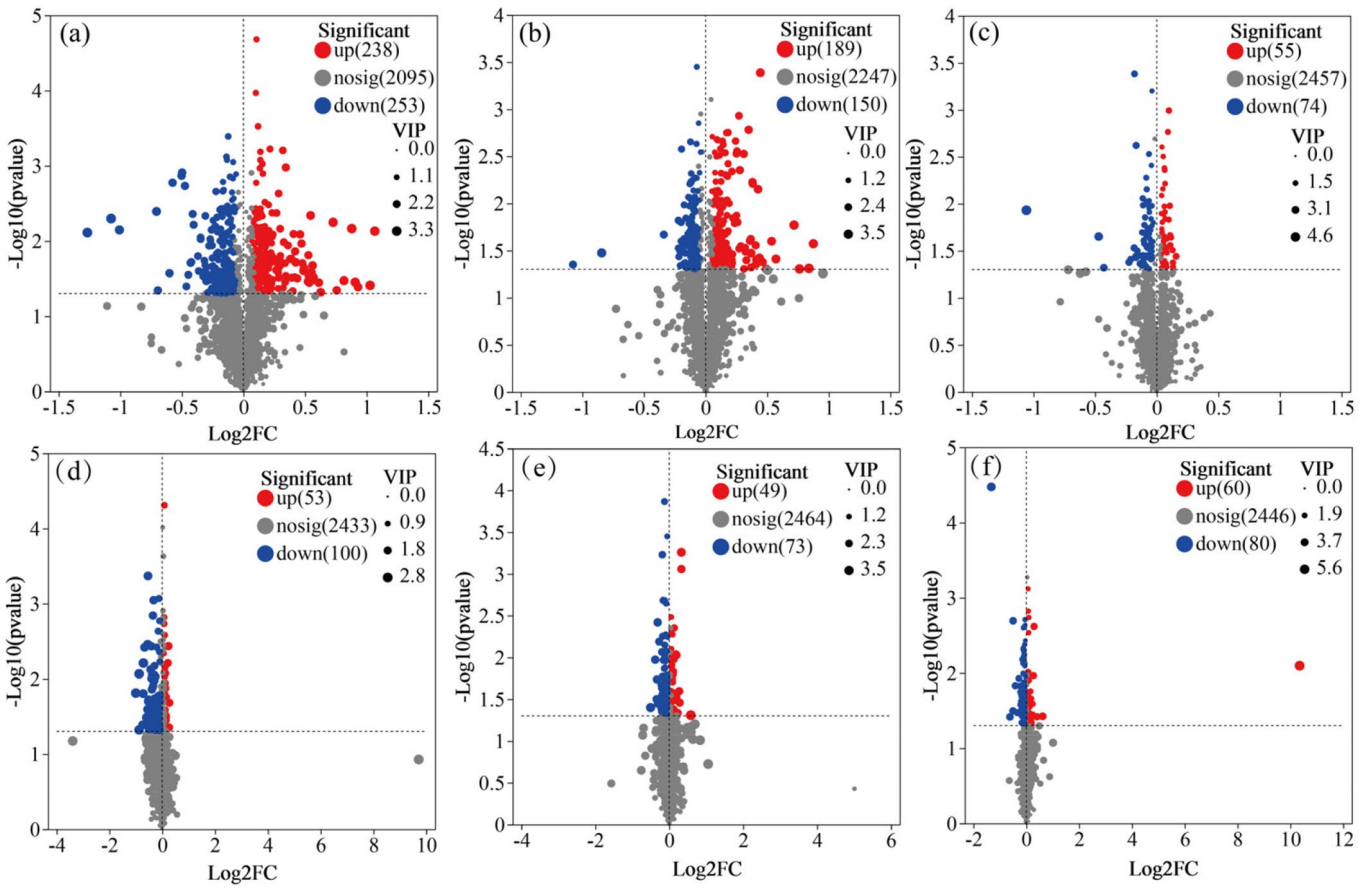
Volcano distribution maps. The symbols of (a), (b), and (c) represent YXY586 under the treatment concentrations of 5, 10, and 15 g/L Zn‐FA, respectively; and (d), (e), and (f) represent GXY703 under the treatment of 5, 10, and 15 g/L Zn‐FA, respectively.

As shown in Figure 5, in the YXY586 variety, compared to control, 491 differential metabolites were significantly altered after spraying 5 g/L Zn‐FA (238 were upregulated and 253 were downregulated). A total of 339 metabolites were significantly altered after spraying 10 g/L Zn‐FA (189 were upregulated and 150 were downregulated), and 129 metabolites were significantly altered after spraying 15 g/L Zn‐FA (55 were upregulated and 74 were downregulated). Similarly, in the GXY703 variety, 153 metabolites were significantly altered after spraying 5 g/L Zn‐FA (53 were upregulated and 100 were downregulated), followed by 122 significantly altered metabolites after spraying 10 g/L Zn‐FA (49 were upregulated and 73 were downregulated), and 140 metabolites (60 were upregulated and 80 were downregulated) were significantly altered after spraying with 15 g/L Zn‐FA.

In the YXY586 variety, after 5 and 10 g/L treatments, the upregulated metabolites were primarily phenolic (including flavonoids) and their derivatives, while the downregulated metabolites mainly included terpenes, lipids, organic acids, and their derivatives. After the 15 g/L treatment, lipids and terpenoids were primarily upregulated, while phenolic (including flavonoids) and their derivatives, organic acids, and their derivatives were primarily downregulated. Similarly, in the GXY703 variety, after 5 and 10 g/L treatments, the upregulated metabolites were mainly phenolic (including flavonoids) and their derivatives, while downregulated metabolites mainly included terpenoids, organic acids, and their derivatives, and lipids. Following the 15 g/L treatment, terpenes and lipids were primarily upregulated, while flavonoids, carbohydrates, and their derivatives were primarily downregulated.

**FIGURE 6:**
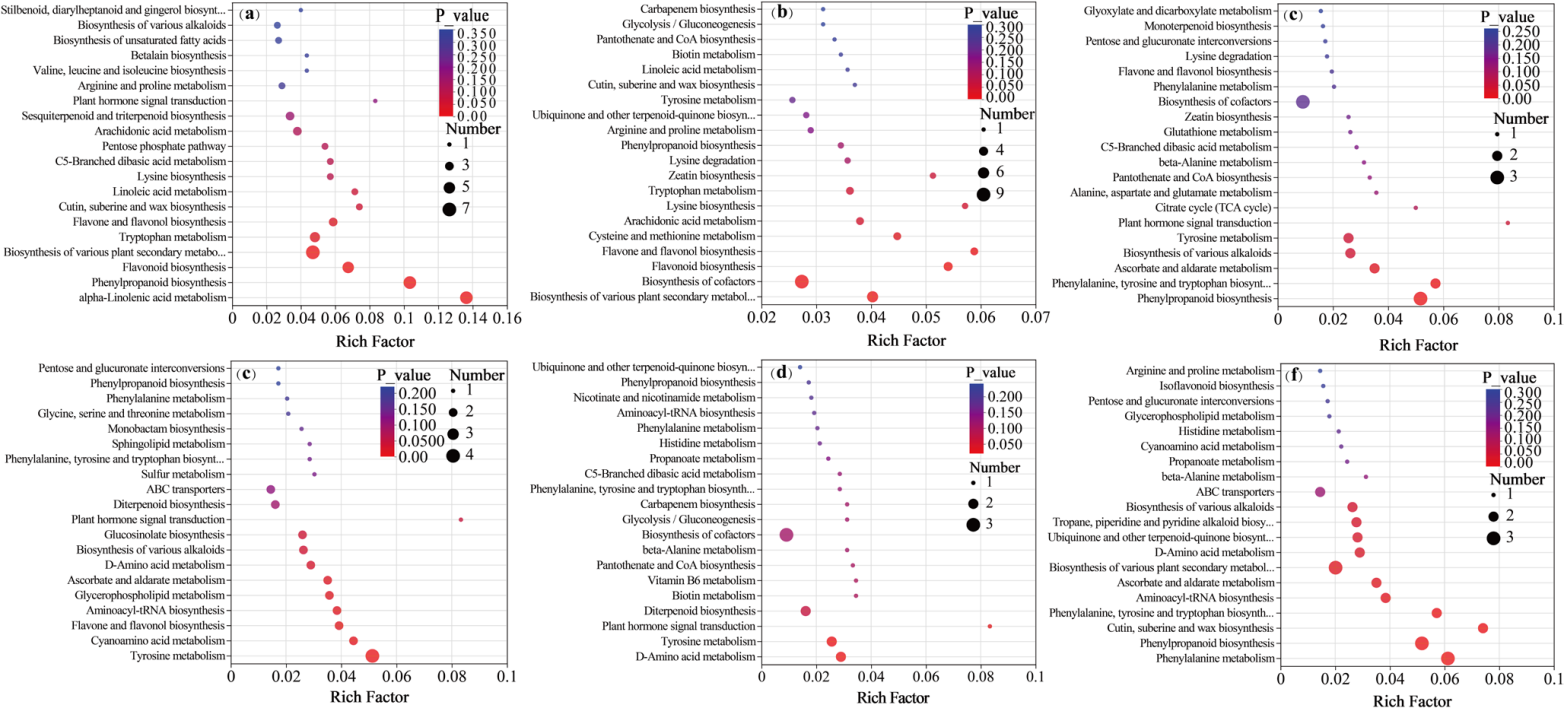
Metabolic pathway analysis of the differential metabolite KEGG. The symbols of (a), (b), and (c) represent YXY586 under the treatment concentrations of 5, 10, and 15 g/L Zn‐FA, respectively; and (d), (e), and (f) represent GXY703 under the treatment of 5, 10, and 15 g/L Zn‐FA, respectively.

The KEGG pathway enrichment analysis results of differential metabolites are shown in Figure 6. In the YXY586 variety, 40 metabolic pathways were identified in the control versus 5 g/L Zn‐FA group, with significant effects (*p* < 0.05) observed in α‐linolenic acid metabolism, phenylpropanoid biosynthesis, flavonoid biosynthesis, flavone and flavonol biosynthesis, and tryptophan metabolism. The largest number of metabolic pathways was identified in the control versus 10 g/L Zn‐FA group, with a total of 42 metabolic pathways identified. Among these pathways, biosynthesis of cofactors, flavonoid biosynthesis, flavone and flavonol biosynthesis, cysteine and methionine metabolism, and arachidonic acid metabolism were significantly affected (*p* < 0.05). In the control versus 15 g/L Zn‐FA group, a total of 24 metabolic pathways were identified, including phenylpropanoid biosynthesis, phenylalanine, tyrosine, and tryptophan biosynthesis, ascorbate and aldarate metabolism, biosynthesis of various alkaloids, and tyrosine metabolism, which were significantly affected (*p* < 0.05). These findings suggest that variations in Zn‐FA concentrations substantially affect the metabolic pathways in rice leaves. The 10 g/L Zn‐FA treatment influenced the most metabolic pathways in rice leaves, indicating that this concentration exerts the greatest impact on foliar metabolism. Additionally, the biosynthesis of flavone and flavonol, as well as flavonoid biosynthesis, was commonly influenced by both the 5 and 10 g/L Zn‐FA treatments.

In the GXY703 variety, 28 metabolic pathways were identified in the control versus 5 g/L Zn‐FA group, with significant effects (*p* < 0.05) observed in tyrosine metabolism, cyanoamino acid metabolism, flavone and flavonol biosynthesis, aminoacyl‐tRNA biosynthesis, glycerophospholipid metabolism, ascorbate and aldarate metabolism, D‐amino acid metabolism, biosynthesis of various alkaloids, glucosinolate biosynthesis, and plant hormone signal transduction. Similarly, a total of 25 pathways were identified in the control versus 10 g/L Zn‐FA group, with significant effects on D‐amino acid metabolism, tyrosine metabolism, and plant hormone signal transduction (*p* < 0.05). As for the control versus 15 g/L Zn‐FA group, 27 pathways were detected, with significant effects (*p* < 0.05) on phenylalanine metabolism, phenylpropanoid biosynthesis, cutin, suberine and wax biosynthesis, phenylalanine, tyrosine, and tryptophan biosynthesis, aminoacyl‐tRNA biosynthesis, ascorbate and aldarate metabolism, D‐amino acid metabolism, ubiquinone and other terpenoid‐quinone biosynthesis, and tropane, piperidine, and pyridine alkaloid biosynthesis. These results indicate that varying concentrations of Zn‐FA had different effects on the metabolic pathways in rice leaves. Additionally, tyrosine metabolism and D‐amino acid metabolism were the common metabolic pathways influenced by both the 5 and 10 g/L Zn‐FA treatments.

## 4 Discussion

The results indicate that Zn‐FA effectively reduces Cd concentrations in rice grains of both varieties to varying extents, with the most significant reduction in Cd concentration observed at a concentration of 10 g/L. However, the impact on Cd content in other tissues varied. Previous studies suggest that genetic differences among rice varieties, as well as the concentration of Zn applied, may influence Cd distribution across different tissues (Zhen et al. 2021; Zhao et al. 2021). Studies have shown that foliar application of Zn fertilizers reduces the heavy metal content in the edible parts of crops such as rice and wheat (Zheng et al. 2023; Saifullah et al. 2016; Yang et al. 2011).

This effect is attributed to the similar chemical properties, uptake pathways, and transport mechanisms of Zn and Cd. As a divalent cation, Zn shares its transport system with Cd. When both are present, they compete for binding sites on transporters; high concentrations of Zn may bind more tightly to transporters, thereby reducing Cd transfer efficiency to the endosperm while increasing Zn transfer (Zheng et al. 2024), such as through transporters *OsIRT1*, *OsHMA2*, *OsHMA3*, and *OsZIP7*. Upregulation of *OsIRT1* expression enhanced Zn uptake in rice, while reduced *OsHMA2* expression lowers Cd concentrations in the xylem. *OsHMA3* regulates rice xylem Cd transport by mediating vesicular segregation of Cd in root cells, whereas *OsZIP7* facilitates xylem loading in roots and intervascular transfer in nodes, preferentially delivering Zn and Cd to developing tissues and rice grains (Zheng et al. 2023; Hu et al. 2019).

Furthermore, foliar Zn can reduce glutathione (GSH) concentration in flag leaves and stems, decrease the expression of Zn transporter genes, and limit Cd transport in the xylem, thereby promoting Cd fixation in other plant parts and ultimately reducing Cd content in rice grains (Zheng et al. 2023). Additionally, foliar Zn application inhibited Cd‐induced increases in hydrogen peroxide (H_2_O_2_) and malondialdehyde (MDA), increased superoxide dismutase (SOD) and catalase (CAT) activities, and mitigated oxidative damage. These improvements enhance the growth and photosynthetic characteristics of the plants, thus alleviating the damage caused by Cd stress (Faizan et al. 2021). Similar to this study, foliar spraying of 0.5% Zn‐EDTA can significantly reduce the Cd content in the grains, probably because Zn‐EDTA inhibited Cd uptake by the rice root system and increased the Zn accumulation in the rice grains, thereby decreasing the Cd translocation to grains (Wang et al. 2020). Additionally, Wang, Li, et al. (2023) reported that applying FA can significantly mitigate the adverse impacts of Cd stress on corn seed germination and seedling growth. The reduction in Cd content by Zn‐FA may be due to its abundance of functional groups, such as aliphatic, hydroxyl, amide, quinone, ketone, and carbonyl groups, which can react with metal ions to form stable complexes, thus reducing Cd content in plants (Song et al. 2023). Consequently, foliar Zn‐FA application appears to be a promising strategy to reduce Cd accumulation in rice grains, likely due to the synergistic effects of FA and Zn. The present study was conducted only on soils in Guizhou Province, and the differences in physicochemical properties of different soils may significantly affect the effectiveness of Zn‐FA application; therefore, it is necessary to follow up with potting trials to compare multiple soil types. For the two varieties, the Zn‐FA application resulted in an increase in Zn content in rice grains. This effect is consistent with previous research showing that foliar application of Zn effectively enhances Zn content in crops. For example, Duan et al. (2018) reported a 90.5% increase in Zn content in rice after the foliar application of ZnSO_4_. Additionally, our findings indicate a positive correlation between the concentration of foliar‐applied Zn and the Zn content in rice grains (Figure 2a). This trend aligns with the results reported by Zheng et al. (2023), where Zn content increased by 93% and 172% in rice grains under the treatments with 0.2% and 0.4% Zn, respectively. Studies have shown that foliar‐applied Zn is efficiently absorbed by the leaves and is transported to the grains, thereby enhancing Zn accumulation in grains (Ahmad et al. 2025). After Cd is absorbed by the root system, it is translocated to the stem via the xylem, facilitated by transporters such as OsHMA2 and OsHMA3. Cd primarily accumulates in the grains via the phloem, translocating from the stem to the grains (Wiggenhauser et al. 2021; Zhong et al. 2021). Different rice varieties exhibit varying capacities for Cd accumulation and transport in their roots, stems, leaves, and panicles. The stem plays a critical role in transporting mineral elements to various organs, with the upward translocation of Cd notably restricted at the stem nodes (Lu et al. 2023). In this study, foliar application of Zn‐FA reduced Cd translocation from the stem to the grains in both rice varieties, except for the GXY703 variety treated with 5 g/L Zn‐FA, which consequently decreased Cd accumulation in the grains. Zheng et al. (2023) reported that foliar Zn application promotes Cd sequestration within the rice stem by downregulating Zn transporter gene expression within the flag leaf, thereby reducing Cd translocation through the xylem.

This study demonstrates that foliar application of Zn‐FA alters the metabolic profile of rice leaves, particularly by regulating phenolic compounds, including flavonoids, which are a subclass of phenolics. Phenolics are crucial secondary metabolites required for the synthesis of plant lignin and pigments (Elguera et al. 2012). Plants mitigate the toxic effects of metals at the cellular level by secreting phenolics, which prevent membrane rupture and oxidative damage, processes that can ultimately affect electron transfer and even lead to cell death, functioning as a self‐protection mechanism (Okem et al. 2015). Meanwhile, reports have shown that phenolic compounds can also reduce toxicity in the cytoplasm by chelating or complexing with metals to form precipitated metals. Thus, the antioxidant and metal‐chelating properties of phenolic compounds play a crucial role in protecting plants from metal‐induced stress (Jańczak‐Pieniążek et al. 2022).

In this study, phenolics such as 6‐caffeoylsucrose, isoferuloyl C1‐glucuronide, and dattelic acid were upregulated by 1.14‐, 1.39‐, and 1.29‐fold, respectively, under 5 g/L Zn‐FA treatment in the YXY586 variety. Similarly, 3‐feruloylquinic acid, 4′‐O‐galloyl sucrose, and 6‐caffeoyl sucrose were upregulated by 1.11‐, 1.14‐, and 1.14‐fold under 10 g/L Zn‐FA treatment. However, at 15 g/L Zn‐FA, phenolic compounds were downregulated, indicating that high concentrations of Zn‐FA may suppress phenolic biosynthesis. In the GXY703 variety, 1‐O‐Cinnamoyl‐beta‐D‐gentiobiose was upregulated by 1.07‐fold under 5 g/L Zn‐FA treatment, while dihydroferulic acid, 4‐coumaric acid, and coumaroyl quinic acid were upregulated by 1.04‐, 1.12‐, and 1.22‐fold under 10 g/L Zn‐FA treatment. Monoferuloyl tartaric acid ester was downregulated by 0.95‐fold under 15 g/L Zn‐FA treatment, likely due to the inhibition of its synthesis caused by the high concentration of 15 g/L Zn‐FA.

Among the phenolic compounds, flavonoids, a subclass of phenolics, were significantly affected by Zn‐FA treatment. Flavonoids are naturally occurring polyphenolic compounds, functioning in regulating cell growth and conferring resistance to biotic and abiotic stresses. Under abiotic stress, the synthesis and accumulation of flavonoids is increased (Li et al. 2022). Moreover, studies have demonstrated that flavonoids protect cells from oxidative damage through free radical scavenging, enhancement of non‐enzymatic antioxidant activity, and regulation of redox balance. Additionally, flavonoids exhibit multiple biological activities, such as anti‐tumor, antioxidant, and anti‐inflammatory (Chen et al. 2023; Tang et al. 2023; Lu et al. 2018).

In the YXY586 variety, compared to control, under 5 and 10 g/L Zn‐FA treatment, flavonoids such as Luteolin‐6‐C‐Glucoside were upregulated by 1.44‐ and 1.41‐fold, Quercetin 3‐beta‐laminaribioside was upregulated by 1.34‐ and 1.28‐fold, and eriodictyol was upregulated by 2.10‐ and 1.83‐fold, respectively. Flavonoids are known to chelate heavy metals, and this can enhance the plant's defense mechanisms against heavy metal stress (Keilig and Ludwig‐Mueller 2009). However, at the higher concentration of 15 g/L Zn‐FA, a decrease in flavonoid levels was observed, suggesting that excessive Zn‐FA may inhibit flavonoid synthesis. This concentration‐dependent response highlights the importance of optimizing Zn‐FA application rates to maximize its beneficial effects. Furthermore, in the GXY703 variety, 3′‐methoxyapigenin, a derivative of apigenin, was upregulated by 1.11‐fold under 5 g/L Zn‐FA treatment. Apigenin has been shown to regulate cellular responses to oxidative stress and DNA damage (Sung et al. 2016), further supporting the role of flavonoids in Cd stress mitigation.

In summary, the foliar application of Zn‐FA at optimal concentrations enhances the biosynthesis of phenolic compounds, particularly flavonoids, in rice leaves. Flavonoids, as a key subclass of phenolics, may play a critical role in mitigating Cd toxicity, reducing their translocation, and protecting cellular structures from oxidative damage. This finding not only provides a potential strategy for safer rice production in Cd‐contaminated environments but also reveals the possible mechanism involved, which is through the alteration of phenolic metabolism, particularly the metabolism of flavonoids.

## 5 Conclusions

This study found that foliar application of Zn‐FA not only decreases Cd content in rice grains but also enhances Zn content, thereby improving the nutritional quality of the grains. A concentration of 10 g/L Zn‐FA was identified as the optimal concentration for spraying, with the most significant reduction observed at this concentration, effectively inhibiting the translocation of Cd from stems to grains. Furthermore, Zn‐FA application (5 and 10 g/L) modulates the metabolic profile of rice leaves, particularly by upregulating phenolic compounds, especially flavonoids, which play a crucial role in mitigating heavy metal toxicity. However, excessive Zn‐FA application (15 g/L) showed an opposite effect compared to the 5 and 10 g/L treatments. It may suppress the biosynthesis of these beneficial compounds. Overall, this study not only provides a potential solution for safer rice production in Cd‐contaminated environments but also reveals that Zn‐FA reduces Cd accumulation in rice grains by modulating foliar metabolites through the alteration of phenolic metabolism, particularly the metabolism of flavonoids. Future research should focus on optimizing Zn‐FA application protocols and exploring its efficacy across diverse environmental conditions to ensure sustainable and safe agricultural practices.

## Author Contributions

**Yuhua Yang:** data curation (equal), formal analysis (equal), investigation (equal), software (equal), writing – original draft (equal). **Xianping Yang:** formal analysis (equal), investigation (equal), software (equal). **Zhidong Xu:** investigation (equal), methodology (equal), writing – review and editing (equal). **Qinhui Lu:** data curation (equal), funding acquisition (equal), supervision (equal), visualization (equal). **Qinghai Zhang:** funding acquisition (equal), supervision (equal), visualization (equal).

## Ethics Statement

The authors have nothing to report.

## Consent

The authors have nothing to report.

## Conflicts of Interest

The authors declare no conflicts of interest.

## Supporting information


**Figure S1.** Effect of Zn‐FA application on Zn translocation factors. Root to grain (a), stem to grain (b), leaf to grain (c), stem to leaf (d) for YXY586; root to grain (e), stem to grain (f), leaf to grain (g), stem to leaf (h) for GXY703. CK stands for the control group, and 5, 10, and 15 for Zn‐FA treatment concentrations (g/L), respectively. The letters indicate significant differences between the control and the three treatment groups.

## Data Availability

The data that support the findings of this study are available from the corresponding author upon reasonable request.
