# Supplementary material for: Effects and Physiological Mechanism of Foliar Zinc‐Fulvic Acid Spraying on Cadmium and Zinc Accumulation in Rice (Oryza sativa L.): Insights From Metabolomics Analysis
Source: Food Sci Nutr. 2025 Jun 13;13(6):e70391. doi: 10.1002/fsn3.70391 (PMC12163346; doi:10.1002/fsn3.70391)
Supplement: Supplementary file 1 — Figure S1. Effect of Zn‐FA application on Zn translocation factors. Root to grain (a), stem to grain (b), leaf to grain (c), stem to leaf (d) for YXY586; root to grain (e), stem to grain (f), leaf to grain (g), stem to leaf (h) for GXY703. CK stands for the control group, and 5, 10, and 15 for Zn‐FA treatment concentrations (g/L), respectively. The letters indicate significant differences between the control and the three treatment groups. [file FSN3-13-e70391-s001.docx]

Title: **Effects and physiological mechanism of foliar zinc-fulvic acid spraying on cadmium and zinc accumulation in rice (*Oryza sativa* L.): Insights from metabolomics analysis**

Yuhua Yang ^a, 1^, Xianping Yang ^a, 1^, Zhidong Xu ^b^, Qinhui Lu ^a,^ *, Qinghai Zhang ^a,^ *

^a^ School of Public Health, the key Laboratory of Environmental Pollution Monitoring and Disease Control, Ministry of Education, Guizhou Medical University.No.6 Ankang Road, Guian New Area, Guizhou, 561113, China

^b^ State Key Laboratory of Environmental Geochemistry, Institute of Geochemistry, Chinese Academy of Sciences, Guiyang 550081, China

^*^ Corresponding authors:

Qinhui Lu

E-mail: [luqinhui1993@163.com](mailto:luqinhui1993@163.com)

Qinghai Zhang

E-mail: [zhqh@gmc.edu.cn](mailto:zhqh@gmc.edu.cn)

^1^ These authors contributed equally to this work.


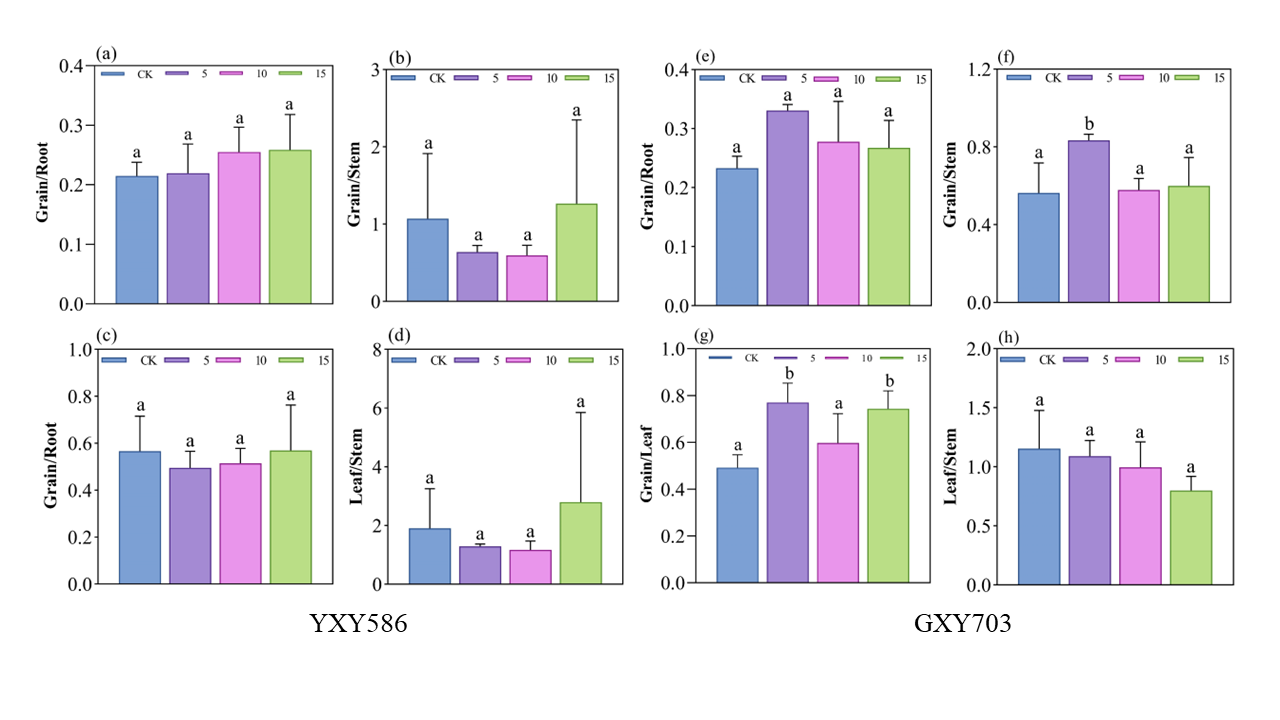


**Figure S1** Effect of Zn-FA application on Zn translocation factors. Root to Grain (**a**), Stem to Grain (**b**), Leaf to Grain (**c**), Stem to Leaf (**d**) for YXY586; Root to Grain (**e**), Stem to Grain (**f**), Leaf to Grain (**g**), Stem to Leaf (**h**) for GXY703. CK stands for control group, and 5, 10, and 15 for Zn-FA treatment concentrations (g/L), respectively. The letters indicate significant differences between the control and the three treatment groups.
